# Biomanufacturing in gene and cell therapy

**DOI:** 10.1016/j.omtm.2024.101261

**Published:** 2024-05-24

**Authors:** Daniel Stone, Xiuyang Wang, Mohamed Abou-el-Enein

**Affiliations:** 1 Vaccine and Infectious Disease Division, Fred Hutchinson Cancer Center, Seattle, WA, USA; 2 Cell Therapy and Cell Engineering Facility, Memorial Sloan Kettering Cancer Center, New York, NY, USA; 3 Department of Molecular Pharmacology, Memorial Sloan Kettering Cancer Center, New York, NY, USA; 4 Center for Cell Engineering, Memorial Sloan Kettering Cancer Center, New York, NY, USA; 5 Division of Medical Oncology, Norris Comprehensive Cancer Center, University of Southern California, Los Angeles, CA, USA; 6 Department of Stem Cell Biology and Regenerative Medicine, Keck School of Medicine, University of Southern California and Children’s Hospital of Los Angeles, Los Angeles, CA, USA; 7 USC/CHLA Cell Therapy Program, University of Southern California and Children’s Hospital of Los Angeles, Los Angeles, CA, USA

## Main text

As of March 2024, the U.S. Food and Drug Administration has approved 36 cell and gene therapy products,^1^ and numerous others are currently under evaluation in clinical trials. This rapidly evolving field offers innovative therapeutic options for patients suffering from severe genetic and acquired diseases. However, as the development and pre-clinical evaluations of these promising new therapies expand, there is a significant need for improved production methods and techniques that are reproducible^2^ and adhere to Good Manufacturing Practices (GMP) standards. This step is critical for assessing the efficacy and safety of these therapies in human trials. Particularly, among the principal challenges in the widespread implementation of gene and cell therapies is the complexity and scalability of the manufacturing processes required for a wide array of products.^3^ Each of these processes requires comprehensive evaluations for efficacy and rigorous analytical quality control. Multiple variables can influence the comparative quality and performance of these products, necessitating that each step in the manufacturing process be individually analyzed. This analysis focuses on its impact on the yield, purity, and therapeutic efficacy of the products, which can be a daunting task.

In response to these challenges, significant advancements and innovations have been made in several key areas to streamline the development and scaling of these therapies. One of the foundational components of gene therapy approaches involve the use of viral vectors, which are bioengineered viruses designed to deliver therapeutic genes into patient cells. The engineering of these vectors is crucial, as it involves the modification of the virus to carry therapeutic genes while ensuring the virus cannot replicate in a way that causes disease or other unwanted side effects. Enhancement of the methods used to produce and purify these vectors has been a major focus for the field with the aim of increasing their safety, transduction efficiency, and scalability. Parallel to viral methods, non-viral gene delivery techniques such as electroporation (using electrical pulses to introduce DNA, RNA, or proteins into cells), lipid-based nanoparticles, and naked DNA or mRNA injections provide alternative pathways that mitigate some risks associated with viral vectors, such as immune activation and potential mutagenesis. Additionally, the development of cell expansion platforms is crucial for producing cell therapies at therapeutic doses. These platforms allow for the growth of therapeutic cells under carefully controlled conditions. Importantly, while it comes at a cost, the integration of automation technologies in biomanufacturing processes is crucial for maintaining consistency, decreasing human error, and enhancing production efficiency.

This special issue of *Molecular Therapy - Methods and Clinical Development* focuses on biomanufacturing in gene and cell therapy. It is dedicated to all aspects of the pre-clinical and clinical manufacturing processes required for these innovative therapies. The issue includes a carefully selected series of review and research articles commissioned by our editorial team. It also features a collection of primary research papers submitted in response to our call for papers on this theme. While each study within this collection has contributed significantly to the field, due to space constraints, we selectively highlight a subset of these studies.

Throughout the history of gene therapy, adeno-associated viral (AAV) vectors have been instrumental due to their good safety profile and efficacy as a gene delivery system. New AAV capsids with designer tissue tropisms are continually being described and validated as therapeutics in pre-clinical models of disease. Simultaneously, AAV vector manufacturing has evolved with significant scientific advancements, focusing on optimizing production processes and enhancing vector quality. In this issue, Mevel et al.^4^ present a significant advancement in AAV-based gene therapy for retinal diseases. By covalently attaching a mannose ligand to amino acids in the AAV capsid, the researchers were able to substantially enhance vector transduction of rat and nonhuman primate retinas. This modification reduces the necessary dosage and minimizes therapy-directed immune responses observed with higher doses. Building on the theme of vector optimization, Mietzsch and colleagues focus on the development of a novel AAV capsid, composed solely of VP3 proteins.^5^ This capsid serves as a high-quality analytical standard for biophysical assays, such as ion exchange chromatography and native mass spectrometry, providing a homogeneous high molecular weight standard. This tool aids in refining analytical methods by ensuring accurate measurement of the physical and chemical properties of AAV vectors, critical for quality control in vector production. Similarly, the study by Marwidi et al.^6^ significantly enhances the production of AAV using a novel baculovirus-based system called the SGMO Helper. Developed to produce AAV6 vectors, this system demonstrates higher yields, better capsid integrity, and greater resistance to proteolytic degradation compared to previous baculovirus-based AAV production methods. Further enhancing analytical capabilities, Heckel et al.^7^ introduce a rapid affinity-based high-performance liquid chromatography method for assessing capsid titer and the full/empty capsid ratio in less than 5 min. This method addresses the complex and time-consuming nature of traditional analytical methods. Last, Rodgers et al.^8^ introduce a droplet digital PCR-based assay for the biodistribution studies of the Smad7 gene therapeutic, AVGN7, utilizing an AAV serotype targeting muscle wasting diseases. This assay enables absolute quantification with high sensitivity and is adaptable for Investigational New Drug applications, bridging a gap in regulatory guidance for gene therapy vector quantification.

Other advancements in viral vector technology, notably through the development and refinement of lentiviral, and retroviral vectors, have been instrumental in advancing T cell therapies for cancer treatment, among other applications. In this issue, Bastone et al.^9^ address critical safety concerns associated with retroviral vectors, known for their risk of insertional mutagenesis which has led to leukemia in some gene therapy recipients.^9^^,^^10^ They developed an *in vitro* genotoxicity assay using murine hematopoietic stem and progenitor cells to detect harmful mutations induced by retroviral vectors, particularly those affecting lymphoid cells. This assay aims to improve safety by identifying potentially oncogenic changes early in the vector development process. Malach et al.^11^ detail the discovery and optimization of a novel small molecule that improves the efficiency of lentiviral transduction in T cells. Through a high-throughput screen of more than 27,000 compounds, six promising candidates were identified and further validated with clinical-grade lentiviral vectors. The lead compound demonstrated enhanced transduction without compromising T cell viability or function and enabled a significant reduction in the required volume of lentiviral vector. In a further attempt to optimize lentiviral vector production, Stibbs and colleagues explore a novel continuous biomanufacturing process for producing lentiviral vectors using a stable producer cell line in a fixed-bed bioreactor.^12^ Their study demonstrates that this system can maintain high-quality vector production over extended periods. This advancement can help address the production bottlenecks previously encountered in the manufacture of lentiviral vectors.

Recent advances in cellular therapies, especially chimeric antigen receptor (CAR) T cell, T cell receptor (TCR), and hematopoietic stem cell (HSC)-based therapies, have shown increasing results in the treatment of various malignancies and genetic disorders. Poletti et al.^13^ explored prostaglandin E2 as a transduction enhancer for HSCs. While it did improve lentiviral vector transduction efficiency, it also reduced the clonogenic potential of human CD34^+^ cells, presenting a disadvantage in repopulation assays. This emphasized the importance of carefully weighing the risks and benefits when selecting critical reagents. Asperti et al.^14^ develop a GMP-compliant process using an integrase-defective lentiviral vector for gene editing of CD4^+^ T cells, enhancing safety and effectiveness in treating hyper-IgM1, a severe immunodeficiency. In a similar vein, Lydeard et al.^15^ employ CRISPR/Cas9 to edit HSPCs for treating acute myeloid leukemia (AML), creating a cell transplant product that reduces the myelotoxic side effects of CD33-targeted therapies. This gene editing shows greater than 70% CD33 modification, maintains cell viability, and exhibits resistance to CD33-targeted therapy. Song et al.^16^ examine the impact of T cell selection methods on the manufacturing of CD22 CAR-T cells. They show that negative selection yielded a higher recovery of CD3^+^ T cells and enriched γδ T cells in the final CAR-T cell product, which might have implications for anti-tumor efficacy and reducing toxicity. Further exploring the optimization of CAR-T cell therapies, Cappabianca et al.^17^ introduce "metabolic priming" in CAR-T cell manufacturing, where T cells activated in a low glucose/glutamine environment show enhanced memory phenotypes and persistence. Yonezawa Ogusuku et al.^18^ develop a GMP-compliant automated process for producing TCR-engineered T cells for AML. By optimizing culture conditions, the team was able to reduce the manufacturing time from 12 to 8 days, achieving high yields of clinically relevant numbers of T cells with an early memory phenotype. The engineered T cells demonstrated specific cytotoxicity against AML in both *in vitro* and *in vivo* models, paving the way for a phase 1/2 clinical trial. As part of ongoing efforts to optimize non-viral delivery systems for CAR T cell therapies, Kitte et al.^19^ compared the efficacy of lipid nanoparticles (LNPs) with electroporation (EP) for delivering CAR-mRNA into T cells. Their findings demonstrate that LNPs provide improvement over EP by ensuring prolonged efficacy of CAR-T cells *in vitro* due to extended mRNA persistence and expression.

We have included a selection of review articles that address critical aspects of manufacturing and regulatory approval for AAV-based therapeutics and CAR-T cell therapies. Blay et al.^20^ explore the development of analytical PCR assays within GMP-compliant AAV manufacturing, detailing the associated technical, biological, and regulatory challenges. The article also proposes future directions for enhancing analytical testing through innovations such as next-generation sequencing and artificial intelligence. Braun et al.^21^ discuss diagnostic assays for detecting pre-existing antibodies against AAV vectors, which are crucial for determining clinical trial participant eligibility. Their work provides a comprehensive overview of the technical and regulatory considerations involved in developing and implementing these assays. Last, Dias et al.^22^ review the impact of the past decade’s advancements on the current CAR-T cell manufacturing landscape, covering the entire process from initial development through to industrial scaling. They highlight the challenges of translating scientific innovations into clinical products and the specific hurdles faced by small academic teams.

In conclusion, the strides made in biomanufacturing for gene and cell therapies, as shown in this special issue, exemplify a dynamic and transformative period in biomedical technology and therapeutic development. As the field continues to evolve, collaboration between researchers, clinicians, regulators, and industry leaders remains paramount to surmounting the challenges ahead and realizing the full potential of gene and cell therapies in revolutionizing patient care. Through continuous innovation and adherence to stringent manufacturing standards, these therapies are emerging as real-world treatments for patients dealing with numerous diseases that were once deemed incurable.
